# AI-Guided DNA-Free and Genotype-Independent Genome Editing for Soybean Improvement

**DOI:** 10.3390/plants15132080

**Published:** 2026-07-03

**Authors:** Hye Jeong Kim, Jia Chae, Seong Ju Han, Jee Hye Kim, Young-Soo Chung, Sivabalan Karthik, Jae Bok Heo

**Affiliations:** 1 Department of Molecular Genetic Engineering, Dong-A University, Busan 49315, Republic of Korea; hjkim83@dau.ac.kr (H.J.K.); cowldk14@gmail.com (J.C.); hsj9587@gmail.com (S.J.H.); kjh48524852@gmail.com (J.H.K.); chungys@dau.ac.kr (Y.-S.C.); 2 Department of Microbiology, Thiagarajar College, Madurai 625009, India; 3 National Center of Excellence in Statistical and Mathematical Modelling on Bio-Resources Management, Thiagarajar College, Madurai 625009, India

**Keywords:** soybean, CRISPR-Cas genome editing, DNA-free genome editing, regeneration reprogramming, genotype-independent regeneration, artificial intelligence (AI)

## Abstract

Soybean is a strategic crop for global protein and vegetable oil supply chains; however, genetic improvement remains constrained by genotype-dependent regeneration, variable transformation efficiency, and regulatory concerns regarding stable transgene integration. This review synthesizes emerging DNA-free and genotype-independent genome-editing frameworks for soybean, where genotype independence is defined as the ability to recover fertile, non-chimeric edited plants across elite germplasm. We critically examine the soybean genome-editing toolbox, including CRISPR-Cas9, Cas12a, multiplex editing systems, base editing, and prime editing, and discuss persistent bottlenecks associated with target selection, off-target assessment, editability, and plant recovery. Particular emphasis is placed on artificial intelligence (AI)-assisted approaches that integrate genomic, epigenomic, chromatin-accessibility, and multi-omics datasets to improve target prioritization, guide RNA design, off-target prediction, and locus- and genotype-specific editability assessment. We further evaluate DNA-free genome-editing technologies, including CRISPR-Cas ribonucleoproteins, transient RNA-based systems, and nanocarrier-mediated delivery platforms, highlighting their potential to generate non-integrative edits while reducing prolonged nuclease exposure. In addition, we discuss regeneration reprogramming strategies based on developmental regulators and morphogenic modules, including BBM-WUS, GRF-GIF, de novo meristem induction, and somatic embryogenesis, as enabling technologies for overcoming cultivar-dependent regeneration barriers. Importantly, this review proposes an integrated AI-to-field framework that connects target discovery, editability prediction, DNA-free editing, regeneration reprogramming, phenotypic validation, and breeding deployment into a unified soybean improvement pipeline. We further highlight emerging opportunities in multi-omics-guided target discovery, genotype-aware prediction models, regeneration-aware editing strategies, and closed-loop machine-learning systems that continuously improve editing decisions through experimental feedback. Collectively, these convergent innovations provide a practical foundation for accelerating the development of climate-resilient, nutritionally enhanced, and industry-ready soybean cultivars.

## 1. Introduction

Soybean (*Glycine max* L.) is among the most widely cultivated legume crops worldwide and serves as a cornerstone of global protein and vegetable-oil supply chains, supporting human nutrition, livestock feed, and a wide range of industrial applications [1]. Its favorable seed composition, high nutritional value, and broad agronomic adaptability have positioned soybean as a strategic commodity crop across temperate and subtropical production regions [1,2]. Global demand for soybeans continues to rise due to population growth, dietary shifts toward plant-based proteins, and expanding industrial utilization, placing increasing pressure on breeding programs to deliver cultivars with stable yield and quality under increasingly variable environmental conditions [1,3,4].

Despite major advances in genomic resources, molecular markers, and breeding technologies, genetic gains in soybean remain constrained by long breeding cycles and the complex quantitative architecture of many agronomic and resistance traits, collectively reducing selection efficiency across environments [4,5,6]. Moreover, domestication bottlenecks and intensive modern selection have narrowed the genetic diversity of elite soybean germplasm, limiting access to novel alleles needed to enhance stress resilience and durable disease resistance [7]. Although transgenic approaches have enabled targeted trait introduction and functional gene validation in soybean [8], their practical deployment is often constrained by regulatory frameworks, public acceptance, and concerns about the stable integration of foreign DNA [9,10]. These limitations have intensified interest in precision-breeding strategies that combine speed, specificity, and regulatory compatibility.

CRISPR-based genome editing provides a programmable, highly versatile means of introducing targeted genetic modifications and has been widely applied in soybean for functional genomics and trait-oriented engineering [11]. The rapid expansion of the genome-editing toolbox, including alternative Cas nucleases, multiplex editing strategies, and next-generation editors such as base and prime editors, has broadened the range of editable loci and improved the precision of allele modification [12,13,14,15,16,17]. However, the routine translation of these technologies into practical soybean improvement remains limited by variable editing efficiency and pronounced genotype dependence.

A major bottleneck arises from the strong dependence of genome-editing outcomes on transformation and regeneration capacity, which varies widely among soybean cultivars and is often exceptionally low in elite, tissue-culture-recalcitrant backgrounds [18]. In addition to transformation efficiency, locus-specific genomic features, particularly chromatin accessibility and epigenetic state, can substantially influence guide RNA activity and DNA repair outcomes, leading to heterogeneous editing performance even within the same genetic background [19]. This persistent disconnect between molecular editing capability and reliable plant recovery represents a major barrier to field-level deployment of genome-edited soybean cultivars [18,20].

To address these challenges, recent research increasingly emphasizes DNA-free and genotype-independent genome-editing frameworks. DNA-free editing approaches, including delivery of pre-assembled CRISPR-Cas ribonucleoprotein (RNP) complexes and transient RNA-based systems, reduce the likelihood of stable transgene integration while minimizing prolonged nuclease activity [21,22]. In parallel, regeneration reprogramming strategies employing developmental regulators and morphogenic modules such as BABY BOOM (BBM), WUSCHEL (WUS), and GROWTH-REGULATING FACTORS (GRF) enable de novo meristem or somatic embryo induction and reduce reliance on cultivar-restricted tissue-culture pipelines [23,24,25]. Together, these approaches signal a shift from incremental protocol optimization toward a system-level redesign of soybean genome-editing workflows.

**Figure 1:**
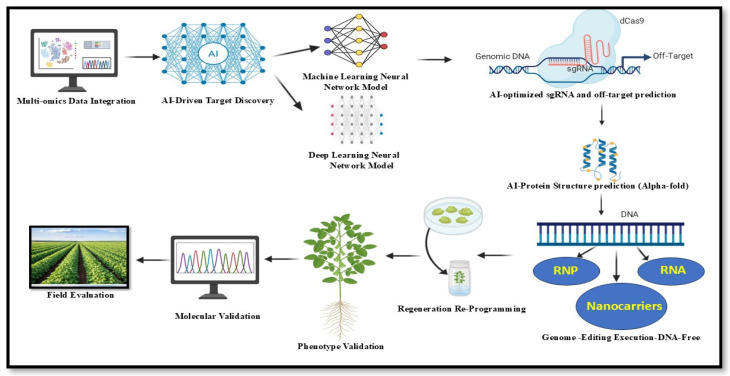
AI-guided framework for DNA-free genome editing in soybean. Multi-omics-based target discovery is integrated with machine- and deep-learning-assisted prioritization for sgRNA optimization, off-target prediction, and protein structure prediction. CRISPR-based editing and DNA-free delivery (RNPs, RNA, nanocarriers) are coupled with regeneration reprogramming to enable genotype-independent recovery. Edited plants are validated through molecular, phenotypic, and field evaluation, supporting efficient translation into soybean breeding. Figure created using BioRender.com.

Concurrently, artificial intelligence (AI) and machine-learning approaches are being integrated into plant biotechnology and precision breeding to address the complexity of target selection and the variability in editing outcomes. Predictive models that incorporate genomic, sequence, and chromatin features can improve target prioritization, optimize guide RNA design, and assess off-target risk [26,27,28]. Beyond guide optimization, emerging AI-driven frameworks aim to predict locus- and genotype-specific editability, thereby reducing empirical trial-and-error and enabling the rational selection of edits with a higher probability of whole-plant recovery [26]. Similar data-driven, high-throughput phenotyping pipelines have already enhanced phenomics and selection efficiency in crop breeding, underscoring their relevance to soybean genome-editing pipelines [29,30]. AI is increasingly emerging as a central analytical layer in genome-editing pipelines, enabling predictive integration of genomic, epigenomic, and phenotypic datasets to guide target prioritization, assess editing feasibility, and inform downstream breeding decisions [26,28,30]. Notably, recent studies have demonstrated the practical potential of AI-assisted genome editing in soybean, where AlphaFold-guided protein structure prediction enabled the rational design of targeted modifications in *GmSWEET10a/10b*, resulting in edited alleles with increased seed oil content accompanied by a reduction in seed protein content under field conditions, highlighting the emerging role of AI in structure-informed functional allele design for crop improvement [31]. In parallel, recent advances in AI-assisted CRISPR workflow design and editability prediction have strengthened the feasibility of predictive genome-editing pipelines [28,32]. Likewise, recent progress in DNA-free genome-editing systems and regeneration reprogramming has further enhanced the practical implementation of genotype-independent genome editing in soybean and other crop species [33,34]. Although previous reviews have examined CRISPR applications, transformation technologies, and regeneration systems [35] separately, an integrated synthesis that explicitly links AI-guided target discovery, editability prediction, DNA-free delivery, and genotype-independent regeneration into a unified soybean improvement framework remains limited. In this context, AI functions not merely as a computational tool for guide design but as a predictive decision layer capable of improving target prioritization, guide design, and editability prediction within genome-editing workflows [26,27,28]. This review presents systems-level AI-guided genome-editing frameworks in soybean that are increasingly structured as integrated, stepwise pipelines that connect in silico target discovery with experimental implementation and breeding translation (Figure 1).

By positioning AI as a central decision-making layer across these stages, the framework enables coordinated optimization of target selection, editing efficiency, and recovery of edited plants. Importantly, integrating DNA-free editing strategies with regeneration reprogramming addresses key bottlenecks in soybean, particularly genotype-dependent transformation constraints, thereby supporting the development of heritable, non-transgenic edited cultivars suitable for breeding applications.

## 2. Genome-Editing Technologies for Soybean Improvement: Capabilities, Bottlenecks, and Emerging Solutions

### 2.1. CRISPR-Cas Systems as the Foundation of Soybean Genome Editing

Within the framework of this review, genome-editing technologies are treated as components of an AI-guided decision system rather than as isolated molecular tools. AI enables prioritizing nuclease systems, predicting locus- and genotype-specific editing outcomes, and selecting editing strategies based on biological feasibility, chromatin context, and regeneration constraints. CRISPR-Cas genome editing has emerged as the dominant platform for targeted sequence modification in soybean, enabling functional genomics and trait engineering with a speed and programmability that conventional breeding cannot match [20,36]. Early soybean demonstrations confirmed that *Streptococcus pyogenes* Cas9 (SpCas9) can generate targeted mutagenesis at endogenous loci, establishing the technical feasibility of genome editing in soybean while simultaneously revealing a persistent limitation: editing outcomes and plant recovery remain strongly genotype-dependent in elite germplasm [11,18].

In parallel, early crop-editing proofs in other species reinforced the broader principle that CRISPR technologies can be translated to complex plant genomes and trait-development pipelines, accelerating expectations that genome editing could transform crop breeding strategies across major agricultural systems and inform precision allele engineering in soybean improvement programs [36]. However, experiences across species also highlighted that successful DNA cleavage alone does not guarantee efficient recovery of edited plants, particularly in crops with recalcitrant transformation and regeneration systems [18,36].

As the field matured, alternative CRISPR nucleases expanded the sequence space amenable to plant genome engineering. Cas12a (Cpf1), which recognizes T-rich protospacer-adjacent motifs and introduces staggered DNA breaks, broadened targeting options and provided architectural advantages for compact guide formats and efficient multiplexing [12,13]. These features are particularly relevant for soybean, where duplicated gene networks frequently necessitate multi-locus perturbation and where delivery constraints become more pronounced under DNA-free or transient-editing objectives [12,13,37].

Despite these advances, a fundamental imbalance has emerged: the molecular genome-editing toolkit, encompassing nucleases, guide architectures, and multiplexing strategies, is advancing more rapidly than soybean transformation and regeneration systems can support the biological throughput. Consequently, even when genome edits are introduced efficiently at the cellular level, the recovery of fertile, non-chimeric edited plants remains inconsistent across elite genotypes. This disparity imposes a practical ceiling on breeding translation that continued innovation in nuclease activity alone cannot overcome [18,20,22].

### 2.2. Precision Genome Editing in Soybean: Base Editing and Prime Editing

Conventional CRISPR editing relies on double-strand breaks followed by endogenous repair, which frequently produces heterogeneous indels that complicate precise allele reconstruction. Precision editors were developed to reduce this uncertainty and enable predictable sequence changes aligned with trait-relevant variants. Base editors enable targeted single-nucleotide substitutions and have been robustly validated in crops, providing a practical route to engineer allele series without complete gene disruption [14,36].

Prime editing extends precision further by enabling templated substitutions, insertions, and deletions through pegRNA-directed “search-and-replace” editing, offering a conceptual pathway to recreate elite haplotypes or correct unfavorable alleles at defined loci [15,16]. However, in soybean, the practical impact of base and prime editing remains limited by delivery constraints and editing efficiency, in addition to cellular competence, including regeneration capacity. Thus, precision editors reduce sequence-level uncertainty but do not automatically resolve genotype-dependent recovery constraints in soybean [18,20,36].

### 2.3. Multiplex Genome Editing and Functional Redundancy in the Soybean Genome

Soybean’s paleopolyploid history has produced extensive gene duplication and functional redundancy, meaning single-gene edits may be buffered by paralog compensation [37]. As a result, multiplex editing is often required to generate strong and reproducible phenotypes, particularly when targeting gene families or layered regulatory networks relevant to stress resilience and seed traits. Multiplex CRISPR toolkits enable simultaneous targeting of multiple loci and have been established in plants for trait stacking and accelerated functional interrogation, making them highly relevant for soybean pathway engineering and combinatorial trait design [17,36].

Nevertheless, increasing multiplex complexity introduces technical and biological trade-offs: larger target sets can reduce per-locus efficiency, increase mosaicism, and expand downstream screening requirements [17,36]. For soybean specifically, multiplex editing can intensify regeneration stress and selection bottlenecks, reducing the likelihood of recovering fertile edited events in complex genotypes, thereby reinforcing the need to couple multiplex designs with regeneration-enabling and genotype-aware frameworks [18,22,38].

### 2.4. Transformation and Regeneration as Persistent Bottlenecks in Soybean Editing

Across soybean genome-editing workflows, transformation and regeneration remain the dominant constraints determining whether editing tools become operational for breeding. The cotyledonary node-based *Agrobacterium*-mediated transformation pipeline is widely used and has been refined over time; however, transformation outcomes still vary substantially across genotypes, explant physiology, and culture responses, thereby limiting reproducibility in elite germplasm [8,39].

Historical soybean transformation studies established the feasibility of stable gene transfer but did not eliminate the central problem: regeneration competence is strongly genotype-dependent and remains a rate-limiting step for routine engineering and trait translation [8,18,39]. Consequently, molecular edits rarely translate into agricultural impact unless transformation and regeneration become substantially more genotype-independent [18,38].

### 2.5. DNA-Free Genome Editing: Technical and Regulatory Considerations

DNA-free genome editing is increasingly prioritized because it reduces the likelihood of stable transgene integration and minimizes prolonged nuclease exposure, both of which can influence regulatory interpretation and downstream event characterization [21,22]. Delivery of pre-assembled CRISPR-Cas ribonucleoprotein complexes represents a foundational DNA-free strategy and provides an effective route for generating targeted edits without introducing DNA constructs [21,22].

However, DNA-free status does not automatically translate into genotype independence. DNA-free delivery still frequently depends on cell type, delivery route, and regeneration pipelines capable of producing fertile plants, thereby maintaining the same transformation and regeneration limitations observed in conventional editing workflows [18,22,25]. Consequently, DNA-free editing can be advantageous for regulatory and biosafety considerations, but on its own is insufficient for broad cultivar deployment unless integrated with regeneration reprogramming and scalable plant-recovery strategies [22,23,24,25]. Regulatory interpretation of genome-edited events should therefore be considered within the framework of applicable national guidance on genome-edited plants [40,41].

Within the broader genome-editing framework discussed in this section, DNA-free approaches represent a complementary strategy rather than a standalone solution. Accordingly, Section 4 examines the principal DNA-free delivery technologies, including RNP complexes, RNA-based systems, and nanocarrier-mediated approaches, and evaluates their practical implementation and translational potential for soybean genome-editing pipelines.

### 2.6. System-Level Implications for Genotype-Independent Soybean Genome Editing

Taken together, advances in nuclease diversity (SpCas9/Cas12a), precision editing (base and prime editing), multiplex design, and DNA-free delivery have expanded the technical scope of soybean genome engineering [11,12,17,20,22,36]. However, predictable recovery across elite genetic backgrounds remains the dominant translational constraint. This underscores the need to treat editor choice, delivery modality, and regeneration competence as a coupled system rather than independent modules, with the relative capabilities and bottlenecks of current genome-editing platforms summarized in Table 1.

Within this framework, AI is not only a guide-design add-on but also a decision layer that can prioritize high-probability target editor genotype combinations by integrating sequence and chromatin-associated information. Such predictive modelling can reduce empirical iterations and improve the efficiency of genome-editing pipelines [26,27,28,46,47]. This systems-level perspective aligns with emerging soybean genomics roadmap efforts that emphasize integrated omics analysis, predictive target design, genome editing, phenotypic validation, and breeding deployment [4,36].

## 3. AI-Guided Target Discovery and Editability Prediction for Soybean Genome Editing

### 3.1. Limitations of Empirical Target Selection in Complex Soybean Traits

Despite the rapid diversification of genome-editing tools, target selection in soybean genome editing remains largely empirical, relying on candidate-gene approaches, homology inference from model species, or prior functional annotation. While effective for proof-of-concept studies, this strategy is poorly suited to complex quantitative traits such as yield stability, abiotic stress tolerance, and seed quality, which are governed by distributed gene networks rather than single loci [6]. In soybean, extensive gene duplication resulting from paleopolyploidy further complicates empirical target selection, as functional redundancy often masks phenotypic effects following single-gene disruption [37].

In addition to biological redundancy, empirical strategies fail to account for the substantial variability in editing efficiency observed across loci. Experimental studies across plant systems demonstrate that identical CRISPR-Cas reagents can yield markedly different outcomes depending on chromatin accessibility, local DNA methylation, and transcriptional status of the target region [19,33,46]. In soybean, these effects are further influenced by genotype-specific chromatin landscapes and tissue-culture responses, leading to unpredictable editing success when targets are transferred from model cultivars to elite breeding lines [33,47]. These limitations underscore the need for predictive frameworks that integrate molecular context with trait relevance during target selection.

### 3.2. Machine-Learning Approaches for sgRNA Design and Off-Target Prediction

Machine-learning (ML) approaches have emerged as powerful tools for improving CRISPR guide RNA (sgRNA) design by capturing complex, nonlinear relationships between sequence features and editing outcomes, and recent advances highlight the growing role of AI-driven CRISPR optimization frameworks in improving editing accuracy, target selection, and predictive design [48,49]. Early predictive frameworks for sgRNA design focused on sequence composition and position-specific nucleotide features, enabling improved prediction of on-target activity compared with earlier heuristic or rule-based design approaches [49]. Subsequent generations of models incorporated structural features, thermodynamic parameters, and experimentally derived efficiency scores, further enhancing predictive accuracy across diverse genomes [27].

Representative deep-learning models have further improved predictive performance in guide RNA design. For example, DeepCRISPR integrates sequence features with epigenetic context using convolutional neural networks to improve prediction of sgRNA activity and off-target potential. Similarly, DeepCpf1 demonstrated improved prediction of CRISPR-Cpf1 guide RNA efficiency compared with earlier rule-based methods. In plant systems, dedicated tools such as CRISPR-P 2.0 and other crop-oriented guide-design platforms incorporate genomic context, regulatory information, and genome variation to support more accurate guide RNA selection in complex plant genomes [27,28,50,51]. Collectively, these platforms provide complementary capabilities, with Rule Set 2 supporting sequence-based sgRNA activity prediction [49], DeepCRISPR incorporating epigenetic context [28], DeepCpf1 optimizing Cas12a guide design [27], and CRISPR-P 2.0 offering plant-specific guide selection and off-target assessment [50]. Plant-specific sgRNA design frameworks have adapted these approaches to account for plant genome architecture, repair pathway biases, and GC-content distributions, resulting in improved editing efficiency and reduced off-target activity in crops [49,50,51]. Although most training datasets originate from rice, *Arabidopsis*, or mammalian systems, broader cross-system predictive frameworks suggest that models trained on heterogeneous datasets may be adaptable to species with larger and more complex genomes [28,52]. These advances suggest that ML-guided sgRNA design can substantially reduce experimental iteration [49,52] and resource expenditure in soybean genome-editing pipelines [50,51]. In several benchmark studies, deep learning-based models have shown significantly improved predictive accuracy compared with earlier heuristic approaches, enabling more reliable identification of highly active sgRNAs and reducing experimental screening requirements in genome-editing experiments [27,28]. For example, benchmark studies reported that deep-learning-based sgRNA prediction models outperformed earlier rule-based approaches in guide-activity prediction, supporting their practical value for reducing experimental screening during genome-editing design [27,28,49]. Although most models are trained on *Arabidopsis* or mammalian datasets, emerging applications in crops, including soybean, indicate that these approaches can improve sgRNA selection efficiency and reduce experimental screening requirements in crop genome-editing pipelines [27,28,49].

### 3.3. AI-Based Prediction of Locus and Genotype-Specific Editability

Beyond sgRNA optimization, a major unresolved challenge in soybean genome editing is predicting editability, the likelihood that a specific locus can be edited efficiently and regenerated into a fertile plant within a given genetic background. Recent AI-driven frameworks have begun to integrate epigenomic features such as chromatin accessibility, histone modifications, and transcriptional activity to predict locus-level CRISPR responsiveness [28]. By integrating chromatin accessibility data, transcriptional activity profiles, and sequence context, these models can predict locus-specific editing outcomes and guide the prioritization of target loci before experimental validation, thereby improving editing success rates in complex crop genomes such as soybean [33,47,53]. These models move beyond sequence-centric design and directly address biological determinants of editing variability.

Genotype effects are particularly pronounced in soybean, where identical editing constructs often yield divergent outcomes across cultivars due to differences in chromatin organization, stress responses, and regeneration competence [20,33,37]. ML models incorporating genotype-specific epigenomic and transcriptomic data have demonstrated improved prediction of editing outcomes across plant backgrounds, thereby enabling the prioritization of targets that are both biologically relevant and technically tractable [28,53]. This represents a conceptual shift from asking which genes control a trait to identifying which genes can be reliably edited and recovered in elite soybean germplasm.

### 3.4. Multi-Omics Integration for Trait-Linked Target Discovery

AI-guided target discovery is further strengthened by integrating multi-omics datasets that capture the genetic architecture of complex traits. Soybean research has generated extensive genome-wide association studies (GWAS), transcriptomic, proteomic, and metabolomic resources; however, these datasets are often analyzed in isolation and are rarely incorporated directly into genome-editing pipelines [54,55,56,57]. ML models that integrate heterogeneous omics layers can identify regulatory hubs, network bottlenecks, and pleiotropic genes as high-impact intervention points [54,55,56,57].

Integrative AI frameworks that combine GWAS signals with gene co-expression and regulatory networks have successfully prioritized causal genes underlying quantitative traits in crops, outperforming single-dataset approaches [54,55,56,57]. In soybean, such strategies are particularly relevant to drought tolerance and nitrogen-use efficiency (NUE), where trait expression often arises from coordinated network behaviour rather than single-gene effects [54,58]. Coupling multi-omics-driven target prioritization with CRISPR-based perturbation enables a systems-level approach to soybean improvement that aligns molecular editing with breeding-scale outcomes. In soybean, ML-assisted analyses have been used to identify genomic regions and candidate loci associated with seed composition and agronomic traits, illustrating the practical utility of AI-enabled prioritization approaches for crop-specific trait improvement [59]. Together, these advances demonstrate that multi-omics-integrated AI frameworks can move beyond descriptive analyses toward actionable target discovery for genome editing in soybean.

### 3.5. Closing the Loop: From In Silico Prediction to Experimental Validation

A critical requirement for AI-guided genome editing is establishing closed-loop pipelines that link computational predictions to experimental validation. Iterative learning frameworks, in which experimental editing outcomes are continuously fed back into model training, have been proposed to improve predictive accuracy across successive cycles [60]. Early implementations in plant systems suggest that active-learning strategies can progressively reduce experimental failure rates and refine the prediction of editing efficiency [52].

For soybeans, integrating AI-based predictions with high-throughput phenotyping, regeneration assessment, and fertility evaluation will be essential to generate crop-specific training datasets. Importantly, failed editing events, particularly those that stall at regeneration or reproductive stages, provide valuable negative data for refining editability models and for capturing constraints that are invisible at the molecular level [60,61]. Over time, these closed-loop systems could enable predictive genome editing that is reliable not only at the DNA level but also at whole-plant and field-performance scales.

More recently, agentic AI frameworks have extended this concept beyond prediction toward autonomous coordination of genome-editing workflows. For example, CRISPR-GPT integrates large language models, domain-specific biological knowledge, computational design tools, and iterative decision-making modules to support CRISPR system selection, guide RNA design, experimental planning, validation, and data interpretation across the gene-editing pipeline. Such agentic AI systems may further accelerate the transition from predictive genome editing to semi-autonomous, data-driven crop improvement platforms [32].

### 3.6. Conceptual Implications for Soybean Improvement

**Figure 2:**
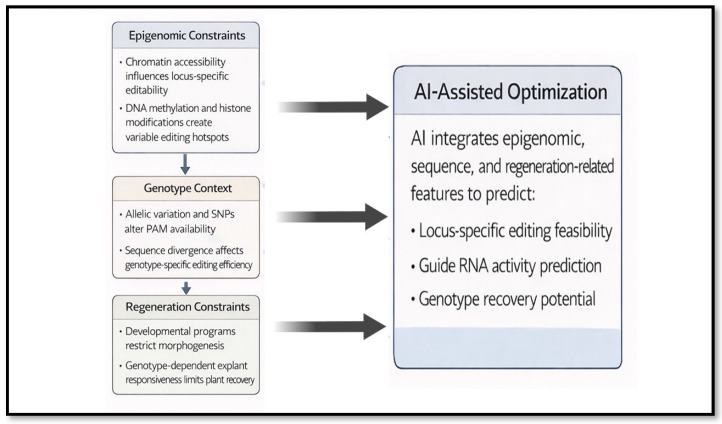
Mechanistic bottlenecks affecting genotype-independent genome editing in soybean. Epigenomic constraints, genotype context, and regeneration limitations influence the feasibility of locus-specific editing and the recovery of edited plants. AI-assisted optimization integrates these biological features to improve target prioritization, guide RNA activity, and predict recovery. Figure created using BioRender.com.

Collectively, AI-guided target discovery and editability prediction redefine genome editing in soybean, transforming it from a trial-and-error molecular intervention into a predictive, data-driven component of an integrated breeding pipeline [6,26,30,62,63,64]. By linking chromatin context, genotype effects, and regeneration competence, AI enables rational prioritization of edits with a higher probability of whole-plant recovery (Figure 2), which schematically integrates literature-supported determinants of soybean editability, including chromatin accessibility, genotype-dependent response, off-target risk, and regeneration competence, into a unified AI-assisted decision framework. This framework encompasses functions ranging from sgRNA optimization and off-target assessment to editability–regeneration coupling and pipeline-level decision support (Table 2). Collectively, these AI-driven approaches enable predictive prioritization of genome-editing targets and establish a decision-support framework linking target discovery, genome-editing design, delivery strategy selection, and regeneration outcomes in soybean genome-editing pipelines.

Recent advances demonstrate that AI can support multiple stages of the genome-editing pipeline, including target discovery, sgRNA optimization, editability prediction, delivery strategy evaluation, and breeding decision support. Deep-learning-based models have improved sgRNA activity prediction and reduced the need for experimental screening, while multi-omics-integrated frameworks have enhanced candidate-gene prioritisation and trait-network analysis [27,28,49,54,55,56,57]. Emerging active-learning systems further enable iterative model refinement using experimental editing outcomes [52,60]. Future research should focus on developing soybean-specific training datasets, integrating regeneration-related parameters into predictive frameworks, and establishing closed-loop AI platforms that connect target discovery, editing, regeneration, phenotyping, and field validation into a unified decision-support system [19,33,60,61].

## 4. DNA-Free Delivery Technologies for Soybean Genome Editing: RNPs, RNA, and Nanocarriers

### 4.1. Rationale for DNA-Free Genome Editing in Soybean

DNA-free genome editing has emerged as a central strategy for accelerating crop improvement while addressing regulatory, biosafety, and public-acceptance concerns associated with stable transgene integration. In soybean, where transformation pipelines are already constrained by genotype dependence and regeneration inefficiency, eliminating stable DNA integration reduces downstream segregation requirements and simplifies molecular characterization of edited events [21,22]. DNA-free approaches are therefore particularly attractive for elite soybean cultivars, where prolonged tissue culture and multi-generation backcrossing are impractical.

From a regulatory perspective, DNA-free editing minimizes the risk of unintended vector backbone integration and persistent nuclease expression, factors that complicate event classification and regulatory assessment in many jurisdictions [41]. Importantly, however, DNA-free status alone does not guarantee regulatory exemption; rather, it reduces the molecular footprint of editing, making downstream regulatory evaluation more tractable when edits resemble naturally occurring or conventionally bred variants [41]. DNA-free delivery can be viewed as an enabling component within a broader precision-breeding framework rather than a standalone solution.

### 4.2. CRISPR-Cas Ribonucleoprotein (RNP) Delivery Platforms

Delivery of pre-assembled CRISPR-Cas ribonucleoprotein (RNP) complexes represents the most direct and widely validated DNA-free genome-editing strategy in plants. RNP delivery enables immediate editing upon cellular entry, followed by rapid degradation of the nuclease complex, thereby reducing off-target risk and mosaicism relative to DNA-based expression systems [21,65,66,67]. Experimental studies across multiple crops have demonstrated that RNP delivery can generate stable, heritable edits without detectable integration of foreign DNA [21,66,67].

In soybean, RNP delivery has been explored primarily using protoplast systems and particle bombardment, which bypass transcriptional and translational constraints [33,38]. While efficient at the molecular level, these approaches are constrained by regeneration capacity, as protoplast-derived regeneration in soybean is highly genotype-dependent and often inefficient [25,38,40]. Consequently, although RNPs offer clear molecular advantages, their translational impact in soybean remains constrained by downstream biological bottlenecks.

### 4.3. RNA-Based and Transient Expression Systems

An alternative DNA-free strategy involves transient delivery of Cas mRNA and sgRNA, enabling short-lived nuclease expression without stable genomic integration. RNA-based systems offer greater delivery flexibility than RNPs and can be introduced via biolistics or polyethylene glycol (PEG)-mediated uptake [22,65].

Transient RNA-based systems can support short-lived expression of editing components without stable genomic integration [22,65]. However, in soybeans, the practical deployment of RNA-based systems remains limited by delivery efficiency, tissue specificity, and compatibility with regeneration. Moreover, transient RNA expression does not fully eliminate the need for tissue culture, underscoring the interconnected nature of constraints on delivery and regeneration [38,40,68].

### 4.4. Nanomaterial-Mediated Delivery Systems

Nanomaterial-mediated delivery has emerged as a promising frontier for DNA-free genome editing, offering the potential to bypass traditional transformation pipelines altogether. Carbon nanotubes, layered double hydroxide nanoparticles, polymer-based nanocarriers, and emerging carbon-dot-based delivery systems have been shown to deliver CRISPR-Cas components or related nucleic acid cargo into plant cells with minimal tissue damage [42,43,44,69,70]. These systems can deliver RNPs or RNA cargo directly into intact tissues, reducing reliance on explant-based transformation.

In theory, nanocarrier platforms could enable genotype-independent delivery across soybean cultivars by decoupling editing from tissue-culture responsiveness. However, current evidence suggests that delivery efficiency, intracellular trafficking, and nuclear localization remain highly variable and species-dependent. In soybean, reproducible nanomaterial-mediated editing has yet to be demonstrated at a scale compatible with breeding pipelines, highlighting the need for further optimization and systematic evaluation [42,43].

### 4.5. Limitations of DNA-Free Delivery in Soybean

Despite their conceptual advantages, DNA-free delivery strategies face several unresolved challenges in soybeans. First, delivery efficiency alone does not guarantee heritable editing; edits must occur in cells that retain regenerative and reproductive competence [25]. Second, DNA-free approaches often require high reagent concentrations or physical delivery methods that can compromise cell viability, exacerbating regeneration bottlenecks in recalcitrant genotypes [33,68].

Third, DNA-free systems do not inherently address genotype-specific differences in chromatin accessibility, DNA repair pathway activity, or stress responses, all of which influence editing outcomes [33,46,53]. Consequently, DNA-free delivery should not be considered a universal solution but rather a component of an integrated genome-editing framework that combines editability prediction with regeneration and reprogramming strategies.

### 4.6. Synergy Between AI-Guided Design and DNA-Free Delivery

The effectiveness of DNA-free genome editing can be substantially enhanced by integrating AI-guided design frameworks. Predictive models that estimate locus and genotype-specific editability can inform not only target selection but also delivery strategy, guiding the choice between RNP, RNA-based, or alternative platforms [26,42,43]. For example, loci predicted to exhibit low chromatin accessibility may benefit from delivery strategies that maximize transient nuclease concentration, whereas highly editable targets may be efficiently modified with minimal RNP exposure [19,33,53].

Moreover, AI-driven optimization of delivery parameters, such as reagent dosage, delivery timing, and tissue selection, could reduce experimental failure rates and improve reproducibility across soybean genotypes [26]. Although direct large-scale validation in soybean remains limited, emerging crop-level studies highlight the potential of AI-assisted strategies to improve target selection and optimize genome-editing workflows. This integration transforms DNA-free delivery from a purely technical challenge into a data-guided decision layer within the genome-editing pipeline.

### 4.7. Positioning DNA-Free Delivery Within Soybean Improvement Pipelines

From a breeding perspective, DNA-free delivery technologies must be evaluated not only by molecular editing efficiency but also by scalability, reproducibility, and compatibility with elite germplasm. While RNPs and transient systems represent important advances, their impact will remain limited unless coupled with regeneration-enabling strategies and genotype-independent recovery systems [23,38].

Within soybean improvement pipelines, DNA-free delivery can therefore be considered an enabling molecular layer that complements AI-guided target prioritization and regeneration reprogramming, rather than a standalone solution. Emerging integrated strategies that combine DNA-free RNP delivery with morphogenetic regulators have also been explored to improve recovery efficiency in recalcitrant genotypes [34]. The comparative strengths, limitations, and deployment value of RNP, RNA, and nanocarrier-based strategies are outlined in Table 3.

## 5. Regeneration Reprogramming, and Genotype-Independent Recovery of Edited Soybean

### 5.1. Why Regeneration Is the True Scalability Bottleneck in Soybean?

In soybean, the rate-limiting step for practical genome editing is rarely the molecular cutting reaction itself; rather, it is the recovery of fertile, non-chimeric plants across elite germplasm. Even when DNA-free delivery achieves high cellular-level editing, edited cells must retain developmental competence and regenerate into whole plants to be useful for breeding, a process that remains strongly genotype- and protocol-dependent in soybean transformation and regeneration systems [8,20,38]. This dependence imposes a structural constraint on translation: the most agronomically valuable cultivars are often the most difficult to regenerate, creating a mismatch between scientific capability and breeding need [20,38].

Regeneration constraints also interact with editing outcomes. Extended tissue culture can increase the risk of somaclonal variation, epigenetic drift, and phenotypic instability during plant regeneration [68,72]. Therefore, “genotype-independent editing” is fundamentally a regeneration problem: scalable soybean genome editing requires precise control of developmental fate across diverse genetic backgrounds, not merely improved delivery or guide design [23,24,25]. Developmental regulators, such as the BBM, WUS, and GRF-GIF modules, can reprogram cell fate and enhance regeneration competence across several crop species, providing promising routes toward genotype-independent transformation systems [23,24]. Consequently, overcoming regeneration bottlenecks will require conceptual frameworks that actively reprogram developmental competence rather than relying solely on incremental improvements to tissue culture protocols.

### 5.2. Conceptual Basis of Regeneration Reprogramming

Regeneration reprogramming aims to redirect somatic cells toward embryogenic or meristematic states by transiently activating developmental regulators that control cell fate. In plants, developmental transitions can be understood as structured changes in transcriptional state that enable competence for organogenesis and meristem formation [25,65,68]. In practice, regeneration bottlenecks persist because standard hormone-driven protocols do not reliably place diverse genotypes into the required competence states, especially under the stresses of transformation and selection [25,61,68].

A key conceptual point is that regeneration reprogramming is not simply “making tissue culture faster”; rather, it decouples transformation success from genotype by converting developmental competence into an engineerable trait. This framing aligns regeneration with genome editing itself, both of which become programmable processes that can be optimized systematically rather than empirically [60,66].

### 5.3. Morphogenic Regulators and Developmental Modules

#### 5.3.1. BBM-WUS and Related Morphogenic Factor Systems

Morphogenic regulators such as BBM and WUS/WUS2 increase regeneration competence by elevating the probability of embryogenic or shoot-forming events from transformed cells. Morphogenic regulator-enabled systems have demonstrated great improvements in transformation outcomes across multiple crop platforms and are increasingly used as conceptual and practical templates to improve regeneration in recalcitrant crops [23,24,34].

For soybean, the translational challenge is not whether morphogenic regulators function in principle, but how to deploy them in a manner compatible with elite cultivar recovery without stable retention of foreign DNA. This requirement directly links regeneration reprogramming to transient or DNA-free editing and to downstream cleanup strategies [34,73].

#### 5.3.2. GRF-GIF Modules and Growth Regulator Strategies

GRF-GIF engineering represents an additional level of enhancement for regeneration. A GRF-GIF chimeric protein can substantially increase regeneration efficiency across multiple crop contexts [24]. Complementary work also shows that GRF family regulators, such as GRF5, can increase transformation competence across diverse plant backgrounds, supporting their use as “pipeline-enabling” regulators rather than trait genes [74].

For soybean, GRF/GIF-type systems are particularly appealing because they can function as temporary recovery accelerators: they facilitate recovery of edited plants but can be removed or silenced during final product development, thereby reducing pleiotropic penalties and supporting “clean-edited” outcomes [24,75].

### 5.4. De Novo Meristem Induction and Tissue-Context Engineering

A complementary paradigm is de novo meristem induction, in which developmental regulators and editing reagents directly trigger meristem formation, reducing reliance on prolonged callus phases. This approach is attractive because it can shorten time-to-plant recovery and reduce genotype dependence by bypassing stages where recalcitrant genotypes often fail [45,76].

In addition, regeneration and editing can be synergistically improved when morphogenic systems increase the fraction of competent cells that both receive editing reagents and undergo rapid regeneration. For example, WUS2-enabled regeneration systems demonstrate increased regeneration capacity and improved CRISPR editing outcomes in rapid regeneration workflows [76], illustrating the principle of regeneration–editing coupling.

### 5.5. Transient Expression, Excision, and “Helper” Constructs for Clean Edited Plants

Because regeneration regulators can induce pleiotropic phenotypes if retained, practical pipelines often require transient expression or post-regeneration removal. Site-specific recombination systems such as Cre/lox provide an established mechanism for excising helper constructs during recovery [73].

For soybean, this design principle is critical for aligning regeneration technologies with DNA-free or low-footprint editing goals: even if morphogenic regulators are used during regeneration, the final product should ideally contain only the intended edit and no persistent helper sequences [34,73].

### 5.6. Editing-Regeneration Coupling: Why “Genotype Independence” Requires Co-Optimization?

A recurring failure mode in soybean genome editing is treating editing and regeneration as separate modules: optimizing guide activity and delivery without ensuring that edited cells can be recovered as fertile plants. Regeneration reprogramming changes the optimization target from edit frequency to edited plant recovery frequency, the breeding-relevant metric [20,60].

Therefore, genotype independence should be framed as a systems engineering outcome produced by coordinated control of: (i) cell entry (delivery), (ii) edit execution (CRISPR chemistry), (iii) developmental competence (reprogramming), and (iv) event cleanup (transient/excision strategies) [33,60,75].

Furthermore, regeneration strategies may require target-dependent optimization. When the edited locus influences developmental regulation, hormone signalling, meristem maintenance, or cellular competence, the effectiveness of regeneration modules may vary with the target gene’s biological function. Therefore, co-optimization of developmental regulators (e.g., BBM-WUS and GRF-GIF), explant source, and regeneration conditions may improve edited plant recovery and overall genome-editing efficiency [23,24,34].

### 5.7. A Practical Roadmap for Soybean: From Recalcitrant Cultivars to Broad Deployment

A realistic soybean roadmap should prioritize regeneration strategies that can be: (1) deployed across multiple elite genetic backgrounds, (2) remain compatible with DNA-free editing systems, and (3) be validated using standardized regeneration metrics (time-to-regeneration, fertility rate, heritability rate, soma-clonal variation burden) [20,60,65]. Multi-genotype benchmarking remains essential because regeneration improvements observed in a single cultivar rarely generalize across soybean breeding pools [20,38].

AI-assisted models could further support this process by integrating transcriptomic, epigenomic, explant-specific, and regeneration-performance datasets to identify soybean cultivars or recipient tissues that are more responsive to BBM-WUS- or GRF-GIF-mediated reprogramming, thereby reducing empirical screening and improving genotype-independent recovery efficiency [28,33,34,53,60]. Ultimately, regeneration reprogramming serves as the enabling layer that translates AI-guided target selection and DNA-free delivery into field-deployable soybean improvement. Without genotype-independent recovery, genome editing remains confined to proof-of-concept demonstrations; with it, editing becomes a scalable breeding technology [20,38]. Accordingly, regeneration reprogramming strategies that decouple plant recovery from genotype, integrate transient or excisable developmental regulators, and align with DNA-free editing objectives form the central enabling layer for scalable soybean genome editing, with key regeneration frameworks and their translational implications summarized in Table 4.

## 6. Priority Trait Classes and an AI-to-Field Genome-Editing Pipeline for Soybean Improvement

### 6.1. Rationale for Trait Prioritization in Soybean Genome Editing

Given the remaining constraints on delivery, regeneration, and genotype independence, the success of soybean genome editing depends not only on technical capability but also on strategic trait prioritization. Traits selected for early deployment must exhibit clear genetic determinism, measurable phenotypes, and substantial agronomic or economic impact, and be compatible with available editing and regeneration pipelines [6,38,41,60]. This prioritization is essential to maximize return on investment and to demonstrate tangible advantages over conventional breeding within realistic timelines [6,38,60].

AI-assisted frameworks can further refine trait prioritization by integrating genomic architecture, environmental relevance, and editability predictions, ensuring that selected targets are not only biologically meaningful but also technically deployable across elite soybean backgrounds [26,33,52]. In this context, priority traits should satisfy three criteria: (i) strong linkage to defined genetic loci or networks, (ii) relevance to climate resilience or market demand, and (iii) compatibility with DNA-free, genotype-independent editing workflows [22,33,38].

### 6.2. Disease and Pest Resistance as First-Wave Targets

Disease and pest resistance traits are highly attractive early targets for soybean genome editing due to their often-discrete genetic control and clear phenotypic readouts. Loss-of-function editing of host susceptibility genes has emerged as a particularly robust strategy for generating durable resistance without introducing foreign resistance genes [78]. In soybean, susceptibility factors associated with fungal pathogens, viruses, and insect pests have been identified through genetic and transcriptomic studies, providing a foundation for targeted editing [78].

AI-guided analytical frameworks can support resistance breeding by assisting the prioritization of candidate susceptibility genes and optimizing sgRNA design based on predicted editability and off-target risk across genotypes [26,28,37,52]. Moreover, multiplex editing enables simultaneous disruption of redundant susceptibility pathways, increasing resistance durability and reducing the likelihood of resistance breakdown [17,78]. These characteristics make disease and pest resistance an ideal proving ground for AI-enabled DNA-free genome editing of soybean. In parallel, beneficial microorganisms can complement host resistance strategies and contribute to integrated crop-protection frameworks [79].

### 6.3. Abiotic Stress Resilience Under Climate Variability

Abiotic stresses, including drought, heat, salinity, and nutrient limitation, pose increasing threats to soybean productivity under climate change [80,81,82,83]. Unlike many biotic resistance traits, abiotic stress tolerance is typically polygenic and context-dependent, complicating conventional breeding efforts [3,84]. Genome editing offers a complementary approach by enabling precise modulation of regulatory genes and signaling pathways that influence tolerance thresholds [36,37].

AI-driven integration of GWAS, transcriptomic, and environmental datasets can identify key regulatory hubs associated with stress adaptation, allowing genome editing to target leverage points within complex networks [26,55,62,63,64]. In soybean, editing transcription factors (TFs) or hormone-response regulators may yield incremental but stable gains in stress resilience when combined across loci [37,84]. Such network-informed strategies align well with AI-guided multiplex design and are compatible with regeneration-reprogramming pipelines optimized for elite cultivars.

### 6.4. Seed Composition and Quality Traits

Soybean seed composition, particularly oil content, fatty acid profiles, and protein quality, is a major determinant of market value and end-use suitability [1]. Many seed-quality traits are governed by well-characterized biosynthetic pathways, making them amenable to precise genome editing [4,85,86,87]. Editing enzymes or regulatory nodes within lipid and protein metabolism pathways can produce predictable compositional shifts without introducing foreign metabolic genes [85,86,87].

AI-assisted modeling of metabolic networks enables identification of pathway bottlenecks and compensatory mechanisms, thereby reducing unintended trade-offs between yield and quality [55,62]. For soybean improvement pipelines, seed-composition traits are attractive because phenotyping is relatively standardized, heritability is high, and edits can often be validated in early generations. These features facilitate rapid translation from laboratory editing to breeding evaluation and commercialization [22,85,86,87,88].

### 6.5. Symbiotic Nitrogen Fixation (SNF) and Nutrient-Use Efficiency (NUE)

SNF and NUE represent a high-impact but technically complex frontier for soybean genome editing. SNF involves coordinated interactions among host genes, microbial partners, and environmental cues. Genome editing offers opportunities to fine-tune nodulation signaling and nitrogen (N) assimilation pathways without disrupting symbiotic balance [36,58,89,90,91]. AI-guided multi-omics integration can identify regulatory nodes that optimize N-fixation efficiency while minimizing carbon costs for the plant [26,58,64,89,90]. Editing such nodes rather than core symbiotic genes may yield incremental improvements compatible with elite germplasm performance. Although SNF traits may not be first-wave targets for commercialization, they are strategically important for long-term sustainability and for reducing dependence on fertilizers.

### 6.6. The AI-to-Field Genome-Editing Pipeline

An effective soybean improvement framework requires integration of AI-guided design, DNA-free delivery, and regeneration reprogramming into a coherent AI-to-field pipeline. In this pipeline, AI models integrate genomic, epigenomic, and phenotypic datasets to prioritize traits and targets with high biological relevance and technical feasibility [26,38,52,62]. This workflow can be conceptualized as a sequential pipeline that integrates AI-assisted target prioritization, sgRNA design, DNA-free delivery, regeneration reprogramming, phenotypic validation, and field-level trait evaluation, thereby linking computational prediction to practical breeding outcomes. Editability prediction further refines target selection by identifying loci likely to be successfully edited and regenerated across elite genotypes [19,28,29,30,31,35,36,37,46].

Selected targets are then subjected to precision genome editing using DNA-free or transient delivery platforms [21,22,40,42]. Regeneration-reprogramming strategies ensure the recovery of fertile edited plants while preserving clean genomes [23,24,25,34,75,76]. Downstream phenotyping and AI-assisted feedback complete the loop, enabling iterative improvement of predictive models [22,26,38,60].

### 6.7. Regulatory and Deployment Considerations

From a translational perspective, the AI-to-field pipeline should align with evolving regulatory frameworks governing genome-edited crops. DNA-free edits that do not involve foreign DNA integration are increasingly evaluated under differentiated regulatory pathways [71]. Clear molecular characterization, predictable edits, and transparent documentation of the editing process are therefore essential components of deployment strategies.

AI-driven traceability systems can assist regulatory dossier preparation by linking edit rationale, molecular outcomes, and phenotypic performance in a reproducible framework [26,62,92,93,94,95]. Aligning early trait targets with favorable regulatory pathways can accelerate adoption among breeders and stakeholders [71,93,94,95].

### 6.8. Toward Scalable and Sustainable Soybean Improvement

Ultimately, the value of genome editing in soybean will be judged by its ability to deliver resilient cultivars at scale, particularly as genome-editing technologies may expand soybean adaptation and support renewed cultivation in underrepresented production regions such as Europe [92]. By integrating trait prioritization with genome-editing design, delivery strategies, and regeneration reprogramming, AI enables a coordinated transition from target discovery to field-level deployment in soybean [54,55,56,57,59,75].

**Figure 3:**
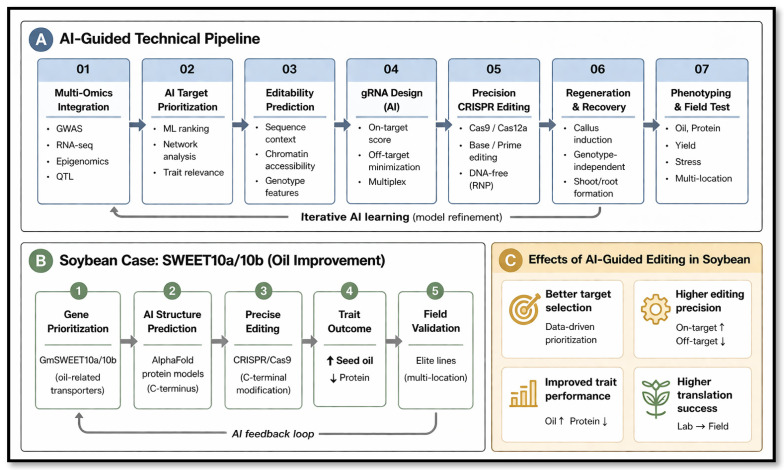
AI-guided genome-editing framework for soybean improvement. (**A**) Sequential pipeline integrating multi-omics target discovery, AI prioritization, editability prediction, gRNA design, precision CRISPR editing, genotype-independent regeneration, and field validation. (**B**) Soybean case of *GmSWEET10a/10b* structure-guided editing, in which AlphaFold-informed modification of functional domains enabled increased seed oil content, accompanied by a reduction in seed protein content under field conditions. (**C**) Effects in soybean, including improved target selection, higher editing predictability, enhanced precision, and efficient lab-to-field translation. Figure created using BioRender.com.

To provide a clear technical representation, this AI-guided framework can be structured as a sequential operational pipeline comprising: (i) multi-omics data integration for target discovery; (ii) AI-driven target prioritization; (iii) editability prediction incorporating sequence, chromatin, and genotype context; (iv) AI-assisted guide RNA design and optimization; (v) precision genome editing using CRISPR-based systems; (vi) genotype-independent regeneration of edited plants; and (vii) phenotypic validation and field-level evaluation for breeding deployment [54,55,56,57,59,68,75]. This stepwise pipeline establishes AI as a central decision-making layer linking molecular design to translational outcomes (Figure 3).

A representative example of AI-guided genome editing in soybean is provided by structure-guided editing of *GmSWEET10a/10b*, where AlphaFold-based protein modeling enabled targeted modification of functional domains, resulting in edited alleles with increased seed oil content accompanied by a reduction in seed protein content under field conditions, reflecting a predictable shift in seed composition associated with altered *SWEET10*-mediated carbon allocation [31,91]. As highlighted in recent analyses, AI-guided CRISPR approaches enable direct modification of protein structure and function rather than simple gene knockout, representing a shift toward precision functional editing in soybean improvement systems [91].

Importantly, the effect of AI-guided genome editing in soybean is reflected in three key aspects: (i) improved prioritization of trait-relevant genes from large multi-omics datasets, reducing reliance on empirical screening [54,55,56,57,59]; (ii) enhanced prediction of editing efficiency and locus accessibility, which is critical in soybean due to genotype-dependent variability in editing and regeneration responses [68,75]; and (iii) increased success in translating molecular edits into stable, field-level phenotypes, including yield- and seed composition-related traits such as oil content [31,91]. Collectively, these findings demonstrate that AI-guided genome editing improves the predictability, efficiency, and translational success of genome editing specifically within soybean breeding systems.

Figure 3 presents a simplified AI-guided technical pipeline for translating soybean genome editing and breeding, in which target selection, DNA-free editing, regeneration competence, phenotypic evaluation, and regulatory considerations are integrated as interconnected translational steps rather than isolated modules.

Together, these advances indicate that AI-assisted genome-editing frameworks are transitioning from conceptual models toward experimentally supported strategies that can enhance precision, efficiency, and scalability in soybean breeding programs [54,55,56,57,59,75,91]. This integrated framework supports more efficient trait validation, facilitates genotype-independent recovery, and enables the development of regulatory-ready soybean cultivars [68,75]. Collectively, these AI-assisted pipelines help bridge the gap between experimental genome editing and scalable crop improvement in soybean.

Within this framework, priority soybean trait classes and their translational bottlenecks are summarized in Table 5, while representative genome-editing case studies are presented in Table 6.

Unlike previous reviews that discuss genome-editing technologies, transformation methods, or regeneration systems independently, this review presents an integrated AI-guided framework that links target discovery, editability prediction, DNA-free delivery, regeneration reprogramming, and breeding deployment within a unified soybean improvement pipeline. By integrating these components into a stepwise decision-support framework, the review provides practical guidance for accelerating the development of climate-resilient, nutritionally enhanced, and genotype-independent soybean cultivars while highlighting future opportunities for AI-assisted precision breeding.

## 7. Conclusions, Challenges, and Future Directions

Soybean improvement has entered a decisive phase in which unprecedented genomic resolution and genome-editing precision now coexist with persistent biological and translational constraints. Over the past decade, advances in sequencing, functional genomics, and CRISPR-based technologies have substantially expanded the scope of genome manipulation in soybeans. However, as this review has highlighted, the principal limitation to real-world deployment now lies less in the availability of genome-editing chemistries than in the predictability, scalability, and genotype-independent recovery of edited plants. Consequently, soybean genome editing must be reframed not as a molecular intervention alone but as a systems-level engineering challenge spanning in silico design, cellular competence, developmental control, and field-level performance. Beyond the technical dimension, the integration of AI-guided target discovery, DNA-free editing, and reprogramming of regeneration also carries important economic and ecological implications, including the potential to accelerate breeding cycles, reduce regulatory complexity associated with transgene-free edits, and improve the sustainability of soybean production systems.

A key conclusion emerging from this synthesis is that regeneration competence represents the primary scalability bottleneck in soybean genome editing. While Cas9/Cas12 platforms, base editors, and prime editors enable increasingly precise DNA modifications, their translational impact remains constrained by genotype-dependent transformation and regeneration. Elite cultivars, which represent the greatest breeding value, are often the most recalcitrant to tissue culture, creating a disconnect between scientific capability and agricultural need. Regeneration reprogramming through morphogenic regulators, growth-factor modules, and de novo meristem induction, therefore, emerges as a foundational enabler rather than a peripheral optimization. Treating developmental competence as an engineerable trait will be essential for converting genome editing into a broadly deployable breeding technology.

Equally important is the growing role of AI in addressing the complexity of soybean trait architecture and the variability in editing. Integrating genomic, epigenomic, chromatin, and phenotypic data into predictive frameworks enables target discovery to move beyond empirical candidate-gene selection toward the rational prioritization of loci that are both biologically impactful and technically amenable to modification. AI-guided editability prediction, therefore, represents a conceptual shift from asking which genes control a trait to identifying which edits can be reliably executed and recovered across elite genetic backgrounds. In this context, AI does not replace biological insight; rather, it amplifies it by reducing empirical iteration and enabling informed decision-making across the genome-editing pipeline.

Despite these advances, several challenges must be addressed to realize the full potential of AI-guided, DNA-free soybean genome editing. Current AI models remain constrained by limited, biased training datasets, particularly by the underrepresentation of elite breeding germplasm and the scarcity of documented unsuccessful editing outcomes. DNA-free delivery systems, while advantageous from regulatory and biosafety perspectives, often suffer from inconsistent efficiency and remain tightly coupled to regeneration bottlenecks. Regeneration reprogramming strategies, although powerful, require precise temporal and spatial control to avoid pleiotropic developmental effects. Addressing these limitations will require standardized benchmarking metrics that extend beyond molecular editing efficiency to include plant recovery rates, fertility, phenotypic stability, and field-level performance.

Looking ahead, the future of soybean genome editing lies in integrating AI-guided design, DNA-free delivery, and genotype-independent regeneration into a closed-loop AI-to-field pipeline. Priority trait classes such as disease and pest resistance, abiotic stress resilience, seed composition, and NUE offer pragmatic entry points for early deployment, providing clear phenotypic endpoints and measurable agronomic value. Over the longer term, stacking and fine-tuning regulatory networks, rather than relying on single-gene interventions, will be essential to deliver stable performance under variable environmental conditions.

To translate this conceptual framework into practical breeding pipelines, several interdisciplinary priorities must be addressed. Expanded multi-omics and genome-editing outcome datasets across diverse soybean germplasm are needed to improve AI-driven target discovery and editability prediction. In parallel, continued innovation in DNA-free delivery technologies should focus on increasing editing efficiency while maintaining minimal genomic footprints compatible with regulatory acceptance. Regeneration reprogramming strategies require improved temporal control and genotype robustness to enable consistent recovery of fertile edited plants. Finally, coordinated benchmarking frameworks integrating editing efficiency, plant recovery rates, phenotypic stability, and field performance will be essential for guiding the scalable deployment of genome-editing technologies in soybean breeding.

In conclusion, the convergence of predictive AI frameworks, clean genome-editing modalities, and regeneration reprogramming represents a paradigm shift in soybean improvement. By transforming genome editing from a trial-and-error laboratory tool into a predictive, scalable, and breeding-compatible platform, these integrated approaches provide a viable pathway toward climate-resilient, nutritionally enhanced, and industry-ready soybean cultivars. Sustained interdisciplinary collaboration among molecular biologists, computational scientists, and breeders will be critical to translate this conceptual foundation into agricultural reality.

## Figures and Tables

**Table 1:** Genome-Editing Technologies for Soybean: Molecular Capability vs. Translational Bottlenecks.

Technology	Editing Principle	Primary Strength	Soybean-Specific Bottleneck	Breeding Relevance	References
SpCas9	DSB–NHEJ	Robust activity	Genotype-dependent recovery	Functional genomics	[11,20]
Cas12a (Cpf1)	Staggered DSB	Expanded PAM	Regeneration ceiling	Multiplex targeting	[12,13]
TALENs	Protein-guided cleavage	High specificity	Low scalability	Gene validation	[11]
Multiplex CRISPR	Multi-locus editing	Redundancy bypass	Mosaicism risk	Polygenic traits	[17,36]
Base editors	Single-base conversion	Predictable alleles	Delivery inefficiency	Allele engineering	[14,36]
Prime editors	Template-driven repair	Highest precision	Low plant efficiency	Haplotype design	[15,16]
CRISPRa/i	Transcriptional control	Reversible effects	Stable expression need	Trait modulation	[17,36]
HDR-based editing	Homology repair	Precise insertion	Extremely rare	Gene replacement	[36]
*Agrobacterium*-mediated delivery	T-DNA transfer	Established pipeline	Cultivar restriction	Trait introgression	[8,39]
DNA-free RNPs	Transient Cas-action	No DNA integration	Recovery limitation	Regulatory ease	[21,22,40]
Nanocarrier delivery	Nanoparticle transport	Bypass transformation	Inconsistent uptake	In planta editing	[42,43,44]
De novo meristem editing	In situ regeneration	Reduced culture	Spatial control	Elite cultivar use	[45]

**Table 2:** AI-Guided Target Discovery and Editability Prediction in Soybean Genome Editing.

AI Function	Data Inputs	Predictive Output	Pipeline Advantage	Representative AI Tools/ Models	References
sgRNA efficiency ML	Sequence features	Activity score	Reduces screening	Rule-set2; Deep CRISPR; Deep Cpf1	[27,28,49]
Off-target prediction	Genome similarity	Risk index	Precision editing	Doench-based scoring; CRISPR-P 2.0; Plant-specific guide-design platforms	[49,50,51]
Chromatin-aware AI	Accessibility/ epigenetics	Editability score	Explains locus effects	Epigenetic- feature CRISPR prediction models	[19,28,33]
Genotype-aware models	Multi-omics	Recovery likelihood	Cultivar compatibility	Integrative ML models combining transcriptomics and epigenomics	[28,33,53]
AI-assisted GWAS prioritization	Trait SNPs	Candidate loci	Trait relevance	ML-assisted GWAS prioritization frameworks	[54]
AI-assisted co-expression network prioritization	Transcriptomics	Regulatory hubs	Network editing	WGCNA-based gene prioritization models	[55,57]
AI-driven multi-omics integration	Genomic layers	Target ranking	Systems view	Multi-omics ML integration frameworks	[56,57]
Transfer learning	Cross-species data	Generalized models	Data scarcity	Cross-species CRISPR activity prediction models	[28,42]
Active learning loops	Edit outcomes	Model refinement	Iterative gain	Adaptive ML training pipelines	[52,60]
Negative-data learning	Failed edits	Constraint mapping	Failure reduction	Failure-aware ML prediction models	[60,61]
Trait-network AI	Graph models	Leverage nodes	Polygenic traits	Graph-based gene prioritization models	[56,57]
Multiplex design AI	Redundancy maps	sgRNA sets	Functional robustness	Multi-target sgRNA optimization models	[28]
Editability and regeneration-aware prediction	Cell competence	Feasibility score	Breeding realism	ML models integrating editing and regeneration parameters	[19,33,61]
Data-guided delivery strategy evaluation	Locus features	Delivery strategy	Improves editing workflow selection	Integrated Editing strategy frameworks	[28,42]
AI-supported breeding decision framework	Integrated datasets	Pipeline prioritization	Translational speed	Integrated breeding Decision support AI systems	[4,30]

**Table 3:** DNA-Free Genome-Editing Delivery Modalities for Soybean.

Delivery Strategy	Cargo	Key Benefit	Critical Limitation	Deployment Value	References
RNP delivery	Cas protein + sgRNA	No transgene	Regeneration dependence	Regulatory-friendly	[21,22]
PEG-protoplast uptake	RNP/RNA	High edit rates	Poor plant recovery	Model testing	[40,71]
Biolistic RNPs	RNP	Genotype- neutral entry	Tissue damage	Elite access	[21]
Cas mRNA systems	RNA	Transient activity	Expression instability	Short exposure	[22]
Carbon nanotubes	RNP/RNA	DNA-free penetration	Variable uptake	In planta promise	[43,44]
Polymer nanoparticles	RNA	Low toxicity	Targeting control	Gentle delivery	[42,43]
Carbon dots	siRNA/sgRNA	Cell-wall crossing	Inconsistent editing	Gene knockdown	[69,70]
In planta editing	RNP/RNA	Minimal culture	Spatial limitation	Rapid cycles	[45]
RNP + morphogenic	RNP + DRs	Improved recovery	Cleanup required	Elite regeneration	[34]
Meristem-targeted delivery	RNP	Reduced genotype effect	Early-stage validation	Scalable concept	[45]
Integrated DNA-free pipelines	Mixed	Regulatory readiness	System complexity	Commercialization	[22,71]

**Table 4:** Regeneration Reprogramming, and AI-to-Field Translation Frameworks for Soybean.

Strategy	Biological Basis	Primary Advantage	Key Risk	Translational Impact	References
Cotyledonary node culture	Organogenesis	Standard recovery	Genotype restriction	Baseline pipeline	[8,39]
Somatic embryogenesis	Embryogenic induction	Whole-plant recovery	Elite recalcitrance	Research use	[25,61]
BBM-WUS modules	Morphogenic TFs	High regeneration	Developmental pleiotropy	Broadening genotypes	[23,24,34]
GRF–GIF modules	Growth regulators	Reduced stress	Expression Tuning	Elite compatibility	[24,74]
De novo meristems	Direct shoot induction	Short Timelines	Spatial control	Fast deployment	[45,76]
Transient DR expression	Temporal activation	Clean edits	Delivery complexity	Regulatory fit	[34,73]
Cre/lox excision	Site-specific recombination	Helper removal	Design burden	Clean events	[73]
Reduced callus duration	Developmental control	Lower somaclonal variation	Optimization need	Phenotype stability	[61,68]
Multi-genotype benchmarking	Comparative testing	True scalability	High resources	Breeding relevance	[38,71]
Editing– regeneration coupling	Systems integration	Plant recovery focus	Pipeline complexity	Field success	[20,60]
AI-guided tissue selection	Developmental state AI	Higher success rates	Data demand	Predictive regeneration	[26,77]
Trait-first deployment	Disease resistance	Clear phenotypes	Trait scope	Early impact	[6,38]
AI-to-field pipeline	Closed-loop learning	Predictable outcomes	Infrastructure	Scalable breeding	[39,60]
Regulatory-ready clean edits	DNA-free + DRs	Faster approval	Policy variation	Commercial release	[3,7,71]

**Table 5:** Priority Trait Classes, Editing Strategies, and Translational Readiness in Soybean Improvement.

Trait Class	Genetic Architecture	Preferred Editing Strategy	AI Contribution	Primary Bottleneck	References
Fungal disease resistance	Susceptibility genes (*S* genes)	Loss-of- function CRISPR	Target prioritization	Regeneration efficiency	[78]
Viral resistance	Host–virus interaction genes	Multiplex knockout	Network analysis	Genotype dependence	[78]
Insect resistance	Defense signaling pathways	Regulatory gene editing	Trait- network modeling	Pleiotropy risk	[78]
Drought tolerance	Polygenic regulatory networks	Multiplex + base editing	GWAS + omics integration	Small effect sizes	[84]
Heat stress tolerance	Transcriptional regulators	Precision modulation	Editability prediction	Context dependence	[84]
Salinity tolerance	Ion homeostasis genes	Allele engineering	Locus ranking	Trade-offs	[84]
NUE	Signaling and transport genes	Fine-tuning regulators	Multi-omics AI	Complex regulation	[58,89,90,91,96]
SNF	Host–microbe networks	Regulatory editing	Network hub detection	Developmental complexity	[58,89,90,91,96]
Seed oil content	Biosynthetic enzymes	Base/prime editing	Metabolic modeling	Pathway compensation	[31,85]
Fatty acid composition	Key desaturases	Precision allele edits	Metabolic flux AI	Yield penalties	[85,86]
Seed protein quality	Storage protein genes	Targeted knockout/ modulation	Trait prioritization	Pleiotropy	[1,31]
Anti-nutritional factors	Single-gene traits	Loss-of-function editing	Editability scoring	Regeneration speed	[87]
Herbicide tolerance	Single enzyme targets	Base editing	Off-target prediction	Regulatory scrutiny	[26,36]
Plant architecture	Hormonal regulators	CRISPRa/i- modulation	Phenotype prediction	Developmental trade-offs	[17]
Yield stability	Highly polygenic	Network-level multiplexing	AI-driven target ranking	Low predictability	[6,38]

**Table 6:** Representative soybean genome-editing case studies showing targeted genes, editing, platforms, mutation outcomes, efficiencies, and phenotypic effects.

Target Gene(s)	Trait Focus	Editing Platform	Mutation Outcome	Editing Efficiency *	Key/ Phenotype Outcome	References
*GmPDS11*; *GmPDS18*	Editing feasibility validation	TALENs; CRISPR-Cas9	Frameshift indels (Loss-of-function)	CRISPR: 26.0–56.7%; TALEN: 20.2–57.7%	Albino phenotype confirming targeted mutagenesis	[11]
*GmCPR5* and endogenous loci	DNA-free functional screening	Cas9 RNP (DNA-free)	Small indels	~4.2–18.1% (protoplast assays)	Rapid mutation screening without DNA integration	[40]
Pooled multiplex targets (multiple loci)	Functional genomics discovery	Pooled CRISPR-Cas9 (multiplex)	Multi-locus indel knockouts	NR ^†^	Mutant populations enabling genotype–phenotype analysis	[97]
*GmFAD2-1A*; *GmFAD2-1B*	High-oleic seed oil	CRISPR-Cas9	Frameshift indel knockouts	Mutations detected in 15/15 T_0_ events ^‡^	High-oleic soybean (~85% oleic acid); transgene-free progeny	[85]
*FAD2-2*	Oil composition engineering	CRISPR-Cas9	Substitutions and indels	~21% mutation efficiency	Increased oleic acid; reduced linoleic acid	[86]
*RS2*; *RS3* (raffinose synthase)	Reduced raffinose oligosaccharides	Multiplex CRISPR-Cas9	1–10 bp indel knockouts	Hairy roots: 41.9–71.0%; T_0_: 50.0–83.3%	Reduced raffinose without a growth penalty	[87]
*RIC1a/2a*; *CLE1A/2A*	Nodulation optimization	Genome editing (reported as genetically optimized lines)	Knockout alleles	NR ^†^	Increased grain yield and seed protein content	[96]
*Gly m Bd* 30K (allergen)	Genotype-independent DNA-free editing	DNA-free CRISPR-Cas9 RNP	Heritable Indels	0.4–4.6% edited plants (E0→E1)	Edited plants recovered without tissue culture; no foreign DNA detected	[71]
Regeneration-enabled targets (multi-locus)	Regeneration acceleration DNA-free	CRISPR-Cas9 + developmental regulators	Heritable Indels	NR ^†^	Improved edited plant recovery across Genotypes Efficient	[34]
Glyma*FAD2-1A*; Glyma*FAD2-1B*	Genome-editing validation	LbCpf1(Cas12a) RNP (DNA-free)	Indels (predominantly deletions)	FAD2-1A: up to11.7%; FAD2-1B: up to 9.1%	DNA-free soybean genome editing with no detectable off-target mutations	[98]

* Editing efficiency is reported as in the original study (e.g., % mutated alleles/events or % edited plants). ^†^ NR, not reported quantitatively. ^‡^ Reported as the number of edited T_0_ events among recovered transformants.

## Data Availability

No new data were generated or analyzed during the preparation of this review article; consequently, data sharing is not applicable.

## Abbreviations

The following abbreviations are used in this manuscript:

AI	Artificial intelligence
ALS	Acetolactate synthase
BBM	BABY BOOM
Cas	CRISPR-associated protein
CRISPR	Clustered regularly interspaced short palindromic repeats
DSB	Double-strand break
ETI	Effector-triggered immunity
gRNA	Guide RNA
GRF	Growth-regulating factor
HDR	Homology-directed repair
HTP	High-throughput phenotyping
INDEL	Insertion/deletion mutation
ML	Machine learning
NHEJ	Non-homologous end joining
PAM	Protospacer adjacent motif
PEG	Polyethylene glycol
PTI	Pattern-triggered immunity
QTL	Quantitative trait locus
RNP	Ribonucleoprotein complex
sgRNA	Single-guide RNA
SNP	Single-nucleotide polymorphism
TF	Transcription factor
WGCNA	Weighted gene co-expression network analysis
WUS	WUSCHEL
